# HIV-1 gp41 and TCRα Trans-Membrane Domains Share a Motif Exploited by the HIV Virus to Modulate T-Cell Proliferation

**DOI:** 10.1371/journal.ppat.1001085

**Published:** 2010-09-02

**Authors:** Tomer Cohen, Shmuel Jaffe Cohen, Niv Antonovsky, Irun R. Cohen, Yechiel Shai

**Affiliations:** 1 Department of Biological Chemistry, the Weizmann Institute of Science, Rehovot, Israel; 2 Department of Immunology, the Weizmann Institute of Science, Rehovot, Israel; NIH/NIAID, United States of America

## Abstract

Viruses have evolved several strategies to modify cellular processes and evade the immune response in order to successfully infect, replicate, and persist in the host. By utilizing *in-silico* testing of a transmembrane sequence library derived from virus protein sequences, we have pin-pointed a nine amino-acid motif shared by a group of different viruses; this motif resembles the transmembrane domain of the α-subunit of the T-cell receptor (TCRα). The most striking similarity was found within the immunodeficiency virus (SIV and HIV) glycoprotein 41 TMD (gp41 TMD). Previous studies have shown that stable interactions between TCRα and CD3 are localized to this nine amino acid motif within TCRα, and a peptide derived from it (TCRα TMD, GLRILLLKV) interfered and intervened in the TCR function when added exogenously. We now report that the gp41 TMD peptide co-localizes with CD3 within the TCR complex and inhibits T cell proliferation *in vitro*. However, the inhibitory mechanism of gp41 TMD differs from that of the TCRα TMD and also from the other two known immunosuppressive regions within gp41.

## Author Summary

HIV uses several mechanisms that allow it to evade immune control, in order to successfully infect, replicate, and persist in the host. Here we report a new mechanism. We utilized bioinformatics and identified a region within the transmembrane domain (TMD) of the envelop proteins of viruses that has high similarity with the α subunit of the T-cell receptor (TCR) TMD. A striking similarity was found within the immunodeficiency virus (SIV and HIV) glycoprotein 41 (gp41). TCR TMDs play an important role in the assembly of the receptor complex composed of the TCR subunits and the CD3 co-receptor chains. We show that a synthetic peptide derived from gp41 TMD co-localizes with CD3 and inhibits T-cell proliferation *in vitro*. Biophysical studies suggest a specific interaction between gp41 TMD and the TMD of the TCRα subunit. Importantly, the inhibitory mechanism of gp41 TMD differs from that of the other two known immunosuppressive regions within gp41. Overall, the present study demonstrates a new weapon that HIV-1 uses to penetrate into the host cell and modulates its immune response. Disassociated from HIV, however, HIV TMD molecule provides a novel mechanism for down regulating undesirable responses and might be used as a new therapy for autoimmune diseases.

## Introduction

The transmembrane domains (TMDs) of the T-cell receptor (TCR) play an important role in the assembly of the receptor complex. The mosaic assembly of the TCR complex, composed of the TCR-α and β subunits and the invariant CD3 co-receptor chains, gives rise to one of the most remarkable and complex receptor structures. A unique characteristic of the multi-protein receptor assembly is the presence of nine potentially charged and conserved amino-acid residues within the transmembrane helices of both the TCR and the CD3 co-receptor [1]–[3]. It has been shown that one basic and two acidic transmembrane residues, within these nine residues, are required for the assembly of each of the three CD3 signaling dimmers (δ-ε, γ-ε, ζ-ζ) with the TCR [4]. In concert with the latter, Manolios *et al* have shown that the stable interactions between the TCRα and CD3 are localized to a short region within the transmembrane domain of TCRα. Moreover, they have shown that the charged amino acids arginine and lysine are essential for this interaction [5]–[7]. In addition, a nine amino-acid peptide derived from the TCRα transmembrane domain, which includes these two charged amino acids (TCRα TMD, GLRILLLKV), interfered and intervened in the TCR function when added exogenously [8].

An important question is whether viruses have exploited this mechanism to interfere with the TCR complex assembly in order to modify T-cell function and thereby evade immune surveillance and enhance viral infection, replication and persistence. Recent studies with HIV support this notion. Similar to other enveloped viruses, HIV requires a fusion of the viral membrane and the cellular membrane to initiate infection. For that purpose it employs a fusion protein, the transmembrane gp41 (gp41) that is non-covalently linked to the surface glycoprotein 120 (gp120). Together, gp120 and gp41 form the enveloped glycoprotein complex, gp160, [9] which is embedded in the viral membrane as a trimmer [10]. The surface gp120 is primarily involved in binding to cellular receptors, whereas gp41 is anchored to the viral membrane and mediates membrane fusion.

**Figure 1:**
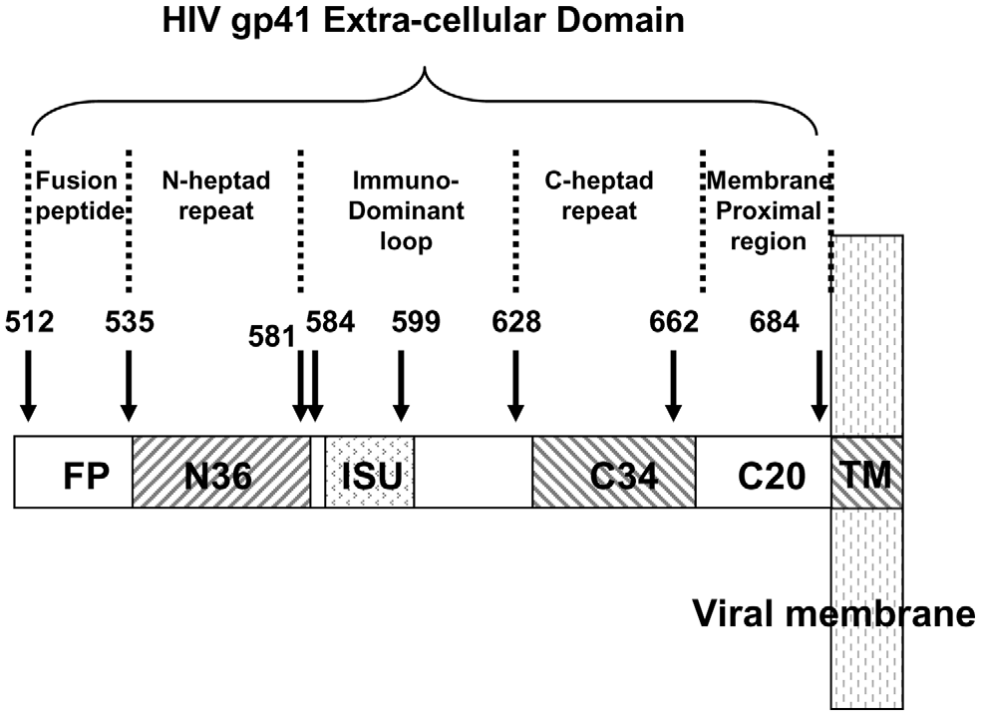
HIV-1 gp41 extra-cellular domain. A scheme showing the functional regions within the extra-cellular portion of gp41 (aa 512–684).

Gp41 is composed of cytoplasmic, transmembrane, and extra-cellular domains [11]–[15]. The extra-cellular domain (ectodomain) contains four major functional regions including a stretch of 16 hydrophobic residues, located at the N terminus, referred to as the “fusion peptide” (FP) [12], [13] (Figure 1). During the initial step of T-cell infection, gp120 binds to the CD4 molecule, which is part of the T-cell receptor complex. The FP then anchors gp41 to the target membrane and initiates the fusion process [15]–[19]. However, it has been shown that anchoring gp41 to the target cell serves not only the purpose of supporting a successful fusion, but also suppresses the ability of the T-cell to be activated and proliferate. FP gains this activity by specifically binding to the TMD of TCRα and interfering with the assembly of the TCR complex; [20]–[23]. Another known immunosuppressive (ISU) region is composed of amino acids 583–599, but its mode of action is not known [24] (Figure 1 and Table 1).

**Table 1:** Designations and Sequences of the Peptides Investigated in This Study.

Designation	sequence
full length gp41 TMD	KKKLFIMIVGGLV**GLRIVFAV**LSIKKK
TCRα CP	GLRILLLKV
AMP - K_6_L_9_	KLKLLKLLKLLKLLK
TAR/PS	KKKMVLGVFALLPLISGSLKK
FP_5–13_ AAAA	GALAAGAAG

Here, we tested *in-silico* a TMD sequence library derived from a group of different viruses and compared it to the TMD of TCRα. The most striking resemblance to the TCRα TMD, including the important arginine residue, was found within the immunodeficiency virus (SIV and HIV) glycoprotein 160 TMD (gp41 TMD). This suggests a role for the TM domain in T-cell suppression as well. To test this hypothesis, we synthesized a peptide comprising the gp41 TM region and examined its immunosuppressive activity and a plausible mode of action. The results indicate that gp41 TMD co-localizes with CD3 within the TCR molecule and inhibits T-cell proliferation *in-vitro*. This effect is specific to the TCR complex, since T-cell activation by PMA/ionomycin is not affected by gp41 TMD. Interestingly, the mechanisms by which the other two immunosuppressive peptides express their activities are different: FP inhibits antigen-specific T-cell proliferation by specifically interacting with the TCRα subunit [23], whereas the ISU inhibits T-cell proliferation induced by anti-CD3 and PMA/ionomycin [24]. Detailed understanding of the molecular interactions mediating the immunosuppressive activity of the gp41 TMD should facilitate the evaluation of its contribution to HIV pathology. Disassociated from HIV, however, the gp41 TMD molecule provides a novel mechanism for down regulating undesirable responses and might be used as an immunotherapeutic tool.

## Results

### Bioinformatics database analysis

**Figure 2:**
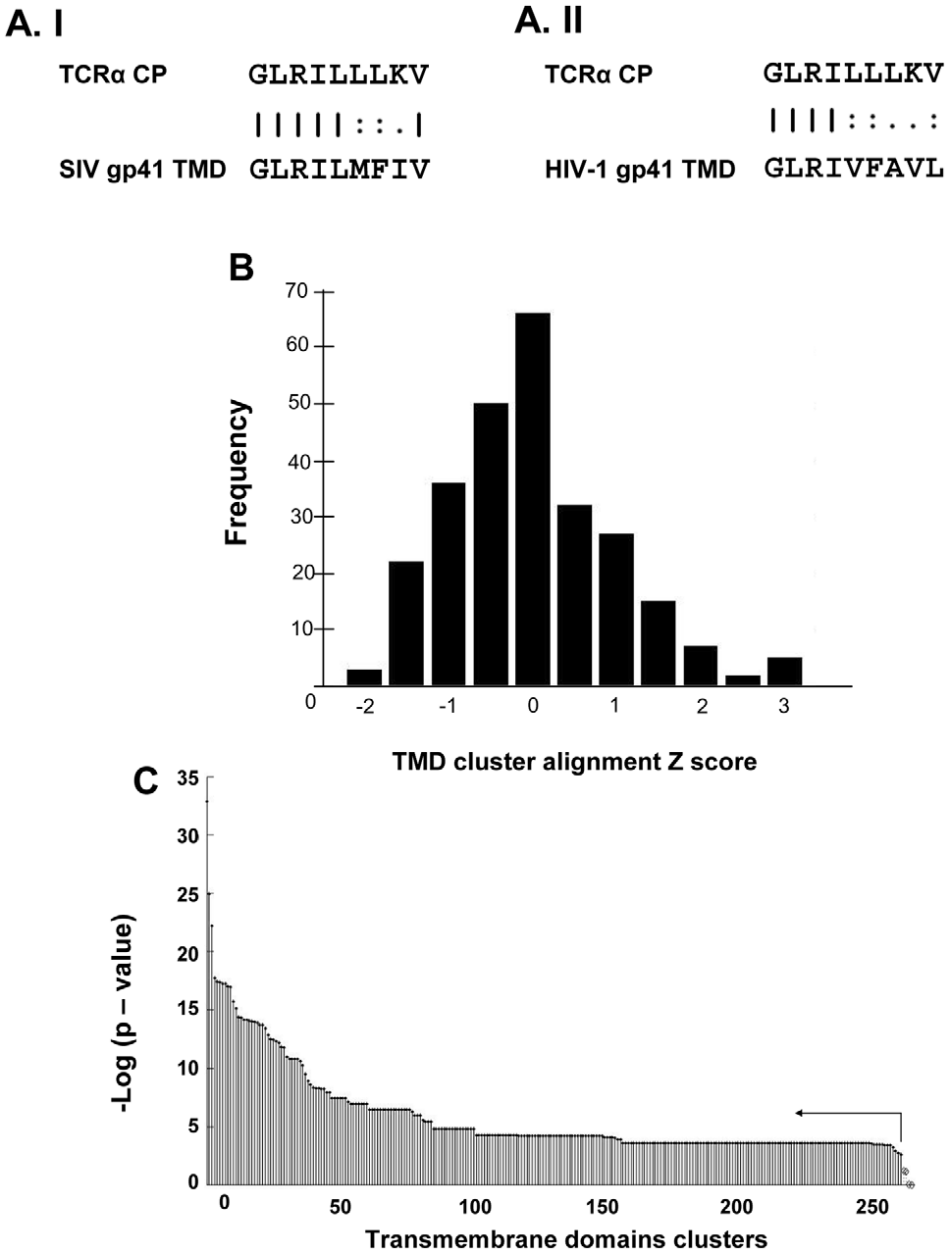
Bioinformatics database analysis. **A**. The sequence alignments obtained from the EMBOSS package: Needle global alignment analysis. **I**) TCRα CP / SIV gp41 TMD sequences alignment **II**) TCRα CP / HIV-1 gp41 TMD sequences alignments. **B**. Z-Score alignment histogram. TMD Entries scores were clustered according to taxonomic species division. The mean of each cluster was Z-normalized. The histogram describes the distribution of alignment scores over the 265 clusters dataset **C**. Wilcoxon Rank Test. The HIV-1 gp41 cluster (n = 47) was compared against all the other 264 clusters using Wilcoxon Rank Test. p-value results of comparisons are presented in a log scale. Significance of ranking was determined according to Benjamini-Hochberg method (E (FDR)<0.05). Clusters which are significantly lowered ranked over HIV-1 gp41 are marked (black dot) and presented left to the arrow. Cluster which were not significantly distinct in ranking from HIV-1 gp41 are marked (diamond).

The nine amino-acid sequence, GLRILLLKV, derived from the TCRα TMD (designated TCRα CP) could interfere with TCR function [8]. In order to examine whether viral protein repertoires contain TMD sequences that are homologous to this immunosuppressive sequence, we took advantage of the Uniport Knowledge Base to construct a viral TMD sequence library. To do this, we performed a systematic pairwise alignment using the EMBOSS package : Needle global alignment [25]. Our results indicated that the top-ranked sequence belongs to the TMD of the SIV gp160 Envelope protein (Figure 2A.I, Entry, accession number: Q8AIH5). This TMD (21 aa) contains a 9 aa sequence with 88.9% similarity and 66.7% identity to the TCRα CP. All the sequence entries (n>3) belonging to a distinct species have been grouped into 265 TMD clusters (over all 2874 sequence entries). Figure 2B displays the averaged normalized Z-score distribution of each cluster. Interestingly, the top-four ranked viral protein clusters were composed of the TMDs of HIV-1 gp160 (namely HIV-1 gp41), Feline immunodeficiency virus (FIV) gp150 and two TMDs within the latent membrane protein 1 (LMP1) from Epstein-Barr virus (EBV). Despite the fact that a single sequence entry within the SIV gp160 cluster (Accession number: Q8AIH5) was the top ranked alignment, the SIV gp160 cluster was ranked lower.

To statistically test the observation that HIV-1 gp41 TMD cluster was significantly ranked higher, a Wilcoxon Rank Test was performed. As shown in Figure 2C, the HIV-1 gp41 TMD cluster was significantly higher ranked than all the other clusters that were not included within this top four group (FIV gp150 and EBV LMP1).

### Gp41 TMD inhibits T-cell proliferation *in-vitro*

Our *in-silico* analysis revealed that four viral protein clusters were significantly ranked in comparison with all the other clusters within our TMD library. As HIV/T-cell interactions are central to HIV infection, we reasoned that these findings may aid in further characterizing the process of HIV infection. The initial step was to examine whether the gp41 TM region, like the TCRα CP, is able to inhibit T-cell proliferation *in-vitro*. For that purpose we prepared lymph node cells from mice immunized with MOG 35–55/CFA (MOG 35–55-immunized) and studied their T-cell responses to the MOG 35–55 peptide. The MOG 35–55 peptide induced strong proliferative responses and cytokine release from T-cells in the draining lymph node cells of MOG 35–55-immunized mice.

**Figure 3:**
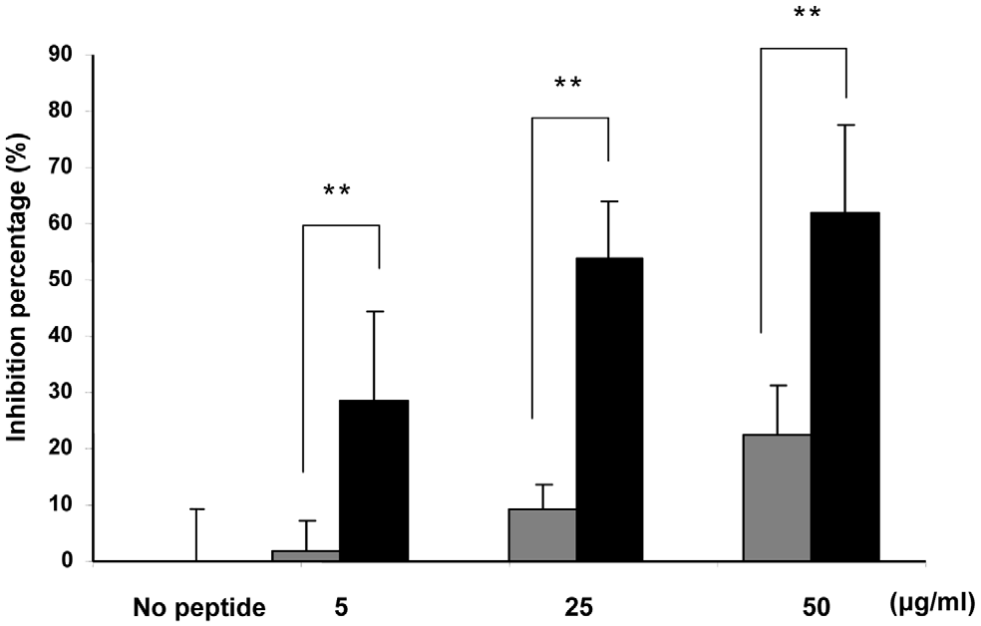
Gp41 TMD inhibits T-cell proliferation *in vitro*. T-cells were activated with the MOG 35–55 peptide and APC in the presence of gp41 TMD (black) or FP_5–13_ AAAA as control peptide (grey) in three different concentrations (50, 25 and 5 µg/ml) and the proliferative responses were assayed. The data are presented as mean inhibitions+SD (*n* = 4 or more, ** *p*<0.01). The uninhibited T-cell proliferative responses were 9382±891 cpm. The background proliferation in the absence of antigen was 232±36 cpm.

We then synthesized a peptide derived from one of the top ranked HIV-1 gp41 TM entries (Figure 2AII, Accession number: P03378, Table 1). Next, we incubated the peptide in the presence of MOG p35–55 antigen with a T-cell line that is specific to MOG p35–55 and determined the proliferative responses after 3 days of incubation, using a H3-thymidine uptake assay. Figure 3 shows that the peptide exhibited dose-dependent inhibition of T-cell proliferation.

The concentration of gp41 TMD in the cell membrane is very small. However, gp41 contains two more immunosuppressant regions (FP and ISU) which probably act synergistically and rapidly with the gp41 TMD. In addition, these regions are directed to the TCR complex after binding of gp120 to the CD4 and co-receptors. In contrast, the gp41 TMD peptide investigated here lacks a receptor, and in addition it is only part of the gp41 complex. This requires much higher concentrations of the peptide to obtain a significant immunosuppressant activity.

### Gp41 TMD inhibits T-cell activation induced by antibodies to CD3

**Figure 4:**
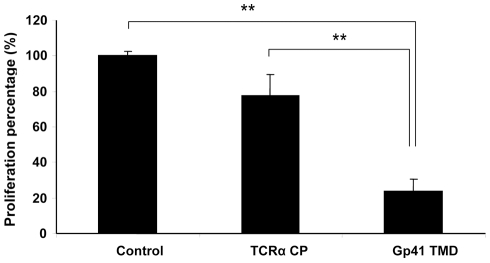
Gp41 TMD inhibit T-cell activation induced by mitogenic antibodies to CD3. T-cells were activated with 1µg/ml anti-CD3 and APC in the presence of 50µg/ml gp41 TMD or TCRα CP and the proliferative responses were assayed. The uninhibited T-cell proliferative responses were 9103±208 cpm. The background proliferation in the absence of antigen was 257±41 cpm. The data are presented as normalized mean proliferation percentage (control, no peptide = 100%) + SD (*n* = 5 or more, ** *p*<0.01). There was no significant statistical difference between the TCRα CP and the control.

To determine whether gp41 TMD can also inhibit T-cell activation other than that induced by APC presentation of specific antigen, we tested the effect of gp41 TMD on T-cell activation induced by a mitogenic monoclonal antibody to CD3. TCRα CP served as a control peptide. Gp41 TMD unlike TCRα CP [8], inhibits the activation of T-cells by mitogenic anti-CD3 (Figure 4). Mitogenic anti-CD3 antibodies activate CD3 signaling regardless of the presence or absence of TCR [22]. These results suggest that gp41 TMD interacts with the CD3, although we cannot rule out other, indirect interactions.

### Gp41 TMD does not inhibit T cell activation induced by PMA/ionomycin

**Figure 5:**
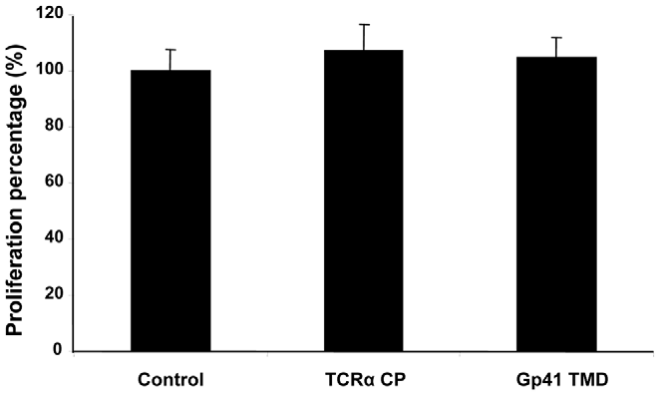
Gp41 TMD does not inhibit T-cell activation induced by PMA/ionomycin. T cells were stimulated with PMA/ionomycin or in the presence of gp41 TMD or TCRα CP, and T cell proliferation was studied. The uninhibited T-cell proliferative responses were 12220±871 cpm. The background proliferation in the absence of antigen was 246±38 cpm. The data are presented as normalized mean proliferation percentage (control, no peptide = 100%)+SD (*n* = 5 or more). There was no significant statistical difference between all groups.

To learn whether gp41 TMD can also inhibit T-cell activation other than that induced by APC presentation of specific antigen or mitogenic monoclonal antibody to CD3 (the TCR complex), we tested the effect of gp41 TMD on T-cell activation induced by PMA/ionomycin. Gp41 TMD, like TCRα CP, did not inhibit the activation of T-cells by PMA/ionomycin (Figure 5). PMA/ionomycin activates the T-cell downstream to the membrane regardless of the presence or absence of the TCR [3]. These findings suggest that gp41 TMD inhibits T-cell activation by interfering with the TCR and CD3 proper function.

### Gp41 TMD co-localizes with the CD3 and TCR

**Figure 6:**
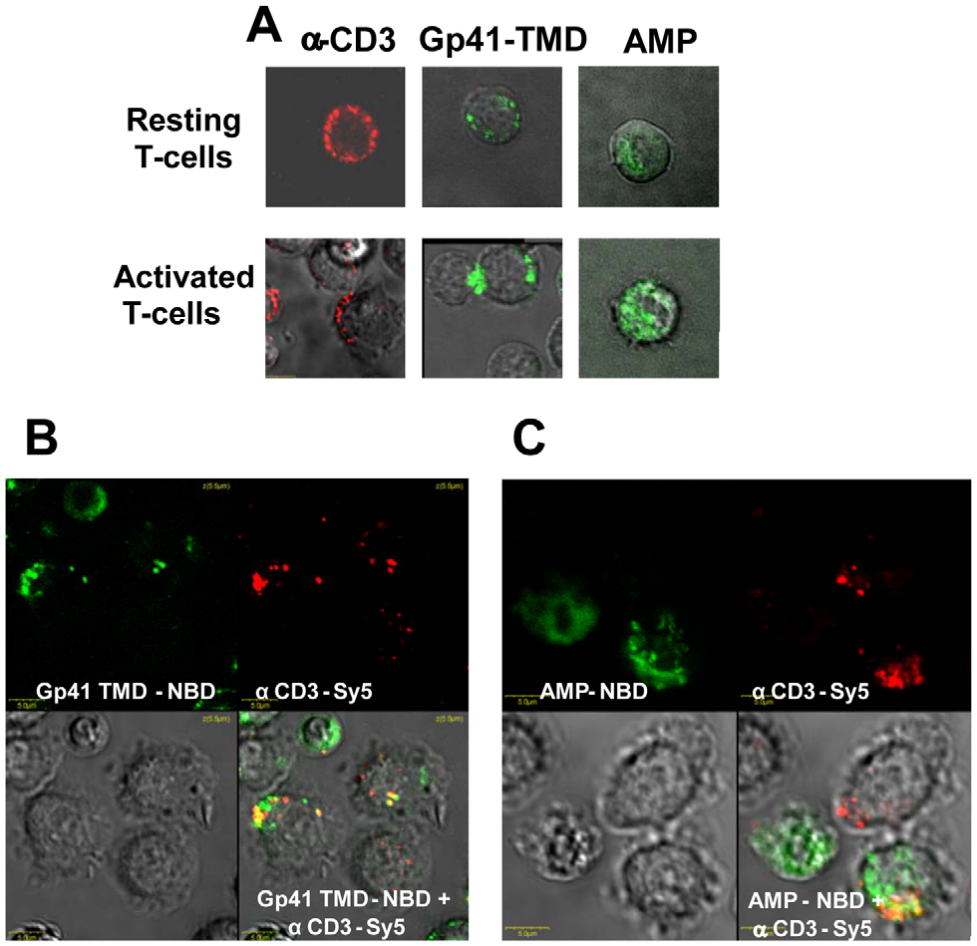
Gp41 TMD co-localizes with CD3 within the TCR complex. Gp41 TMD-NBD and AMP-NBD were used to study peptide binding to the membranes of resting and activated T cells in combination with Sy5-labeled antibodies to CD3. The T cells had been activated by incubation with APC and the MOG 35–55 peptide. (**A**) Distribution of gp41 TMD-NBD, AMP-NBD and CD3 molecules in resting and activated T cells. (**B**) Co-localization of gp41 TMD-NBD with the CD3 molecules. (**C**) Co-localization of AMP-NBD with the CD3 molecules.

We utilized confocal microscopy to further support our findings that the gp41 TMD peptide interacts specifically within the TCR complex and does not home to the cytoplasm. It has been reported that the TCR, CD3, and CD4 receptors, among other components, are localized in micro-domains in the membranes of activated CD4+ T cells [2]. Using Sy5 labeled antibodies to the CD3 molecules, we examined the localization and distribution of the CD3 molecules in the membranes of resting and activated T-cells. In resting T-cells, the CD3 molecules were found in micro-domains all around the T-cell, while the distribution of the CD3 molecules resembled a capping shape in activated T-cells (Figure 6A). In order to examine the distribution of gp41 TMD in the membrane of resting and activated mice T cells, we used gp41 TMD peptide conjugated to the fluorescent probe 4-chloro-7-nitrobenz-2-oxa-1, 3-diazole (NBD; gp41 TMD-NBD). Rather then uniformly labeling the T-cell membrane, the gp41-TMD NBD showed a heterogeneous membrane distribution and a capping shape on the membrane of activated T-cells. This distribution in membrane domains contrasted with that of a control membrane-active amphipathic peptide conjugated to NBD (AMP-NBD), which demonstrated a uniform distribution and no capping shape on activated T-cell membranes (Figure 6A). In contrast to the AMP-NBD control peptide, the gp41-TMD conjugates exhibited the same distribution pattern as the CD3 molecules. In the next step, we demonstrated that the gp41 TMD not only showed the same distribution pattern but also had a significantly higher percentage of co-localization with the CD3 molecules (58.8%, Figure 6B) compared to the AMP-NBD control peptide (27%, Figure 6C) (p<0.05).

### Fluorescence Energy Transfer (FRET) measurements reveal a specific interaction between gp41 TMD and TCRα CP

**Figure 7:**
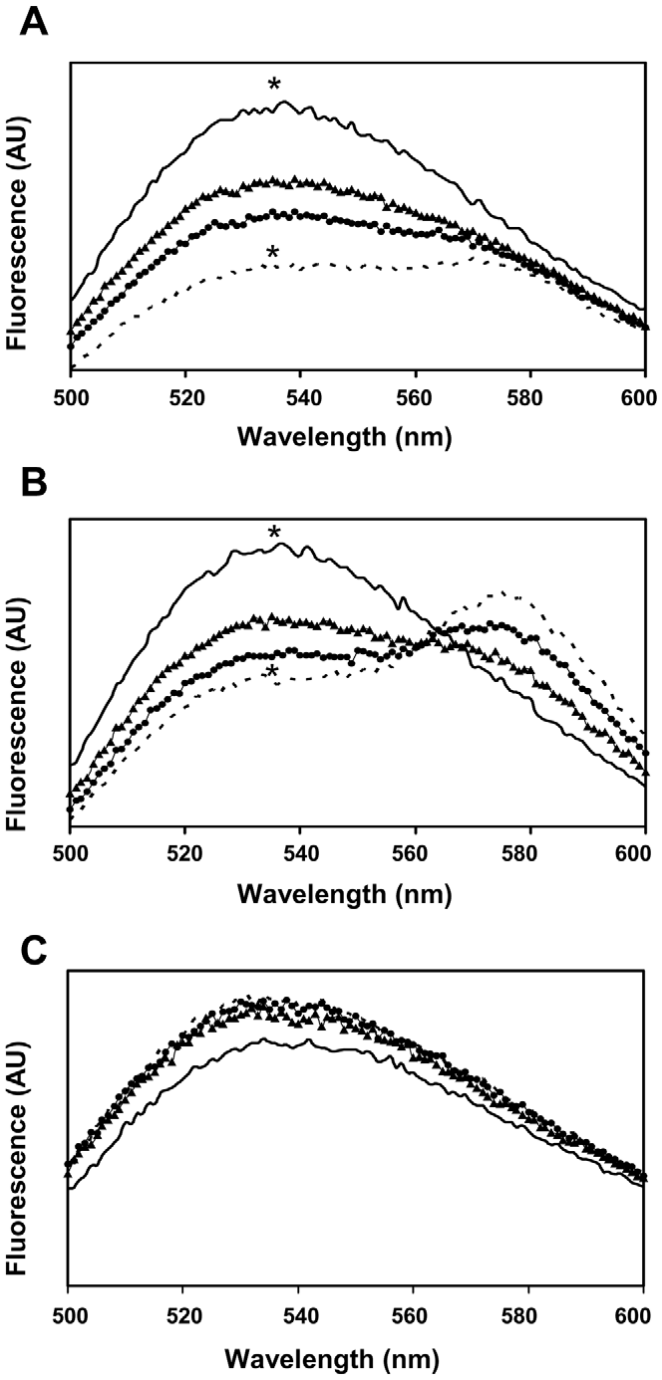
Fluorescence Energy Transfer (FRET) measurements reveal a specific interaction of the gp41 TMD and the TCRα CP. Fluorescence spectra were obtained at room temperature, with excitation set at 467 nm (5-nm slit) and emission scan at 500–600 nm (10-nm slits). NBD-labeled CP peptide was added first from a stock solution in DMSO (final concentration 0.1 µM and a maximum of 0.25% (v/v) DMSO) to a dispersion of PC LUV (100 µM) in PBS. This was followed by the addition of: **A**. Rho labeled gp41 TMD peptide **B**. Rho labeled TCRα CP **C**. Rho labeled TAR/PS TM control peptide. Fluorescence spectra were obtained in four different ratios of Rho-peptide∶NBD-peptide: 0∶4 (black line), 1∶4 (black triangle), 2∶4 (black circle) and 3∶4 (dashed line). The fluorescence values were corrected by subtracting the corresponding blank (buffer with the same vesicles concentration). Statistical analysis was performed at the pick measurements (∼537 nm, n = 5, * p<0.05).

We labeled the TCRα CP peptide with NBD as the donor fluorophore, and both gp41 TMD and TCRα CP peptides with Rho-TAMRA as the acceptor fluorophore. In addition, we also labeled an unrelated transmembrane peptide derived from the *E.coli* aspartate receptor (TAR/PS) as a control hydrophobic peptide (Table 1). We then measured the fluorescence energy transfer (FRET) between fluorescently labeled TCRα CP-NBD peptide and gp41 TMD – Rho (7A), TCRα CP-Rho (7B) or TAR/PS – Rho (7C). The assay was performed in a model lipid environment of large unilamellar vesicles (LUV) composed of PC phospholipids. Four ratios of Rho-peptide∶NBD-peptide were used: 0∶4, 1∶4, 2∶4 and 3∶4. The TCRα CP-NBD peptide showed about ∼50% energy transfer in the presence of the gp41 TMD - Rho peptide at an acceptor-to-lipid ratio of 1∶1000 (Figure 7A), indicating an interaction between the two peptides. When we examined the energy transfer of TCRα CP-NBD peptide in the presence of TCRα CP-Rho peptide, there was lower energy transfer (∼37%, 7B). The TAR/PS – Rho control peptide did not show energy transfer (7C).

## Discussion

During evolution, viruses have evolved various strategies to modify cellular processes and evade immune responses, which allow them to successfully infect, replicate, and persist in the host. Here we utilized bioinformatic tools to identify a new region corresponding to the TMD of the HIV-1 gp41 ENV glycoprotein that enables the virus to inhibit T-cell proliferation. We identified viruses that contain sequences within their TM region that are similar to TCRα CP, an immunosuppressant domain within the TCRα TMD (Figure 2). The results revealed four viral protein TMD clusters, namely, HIV gp160, FIV gp150 and two TMDs within EBV LMP1, that were significantly ranked in comparison with all other TMDs within our library (Figure 2C).

Dukers *et al.* have reported that purified recombinant EBV LMP1 suppress activation of T-cells. They found that a 7-amino acid peptide (LALLFWL) within the TMD1 of EBV LMP1 has immunosuppressant activity [26]. However, this region does not overlap with the EBV LMP1 TMD1 cluster that emerged from our bioinformatic analysis (GLALLLLLL). Interestingly, within our top four highest ranked clusters we identified another EBV LMP1 TMD region within the TMD3 (GLGLLLLMV). However, since HIV/T-cell interactions are central to HIV infection, it was most interesting to investigate whether the HIV-1 gp41 may be exploited by HIV to modulate and interfere with T-cell activation.

Gp41 TMD is one of the most conserved regions within the gp41 sequence [27]. Studies have suggested that this region is involved in many important biological functions such as anchoring of the Env glycoprotein in both viral and cellular membranes [28], cell-cell fusion [29] and in the mixing and fusion of phospholipids between two lipid vesicles [30]. The fact that T-cells are targets of HIV-1 raised the question whether this TMD is also exploited by the virus to modulate the T-cell immune response. Strikingly, we found that a synthetic peptide resembling the gp41 TMD is able to co-localize with the TCR complex and suppress T-cell proliferation *in vitro*, and, therefore, may play a role in infection as an immunosuppressive region.

It has been shown previously that the HIV-1 gp41 has two other immunosuppressive regions: FP [21]–[23] and ISU [24]. The mechanisms by which these two immunosuppressive regions express their activity are different: FP inhibits antigen-specific T-cell proliferation by specifically interacting with the TCRα [23], whereas ISU inhibits T-cell proliferation induced by anti-CD3 and PMA/ionomycin [24].

To better understand the mechanism by which the TM region exerts its immunosuppressive activity, we examined the abilities of the gp41 TM peptide to inhibit T-cell proliferation when triggered through different signal-transduction cascades. Interestingly, we found that gp41 TMD, unlike the FP [21], [23] or TCRα CP [8], inhibits T-cell activation induced by mitogenic antibodies specific for the CD3 molecule (Figure 4). This interaction activates T cells regardless of their TCR and is downstream of the TCR activation pathway. This is despite the fact that this TM peptide has high homology to TCRα CP as found through bioinformatic analysis. To follow the mode of action underlying the latter inhibition, we further examined the interaction between gp41 TMD and TCRα CP by using FRET. The data summarized in Figure 7 reveal a direct interaction between these two TMDs. Note that gp41 TMD did not inhibit T-cell activation triggered by PMA/ionomycin. Overall the data support the notion that gp41 TMD most likely interacts with the TCR complex in a way that interferes with both the TCR and CD3 proper function. Our confocal microscopy results indicate that the gp41 TMD-mediated interference is likely to occur within the cell membrane since the peptide is not detectable elsewhere (Figure 6).

As discussed previously, the immunosuppressive domain (ISU) in gp41 (Figure 1 and Table 1) is capable of inhibiting T-cell activation [24]. Similarly to gp41 TMD, the ISU inhibits T-cell activation triggered by CD3-specific antibodies [24]. However, in contrast to gp41 TMD, ISU can also inhibit T-cell activation triggered by PMA/ionomycine [31]. Thus, ISU simultaneously targets protein kinase C activity [32] and the events related to T-cell activation that occur within the cell membrane [33]. In contrast, gp41 TMD only targets T-cell activation within the cell membrane. Hence, gp41 equips HIV-1 with at least 3 different inhibitors of T cell activity: gp41 TMD, FP and ISU. Each of them uses a different mode of action, which we propose can function at different time points during viral infection.

In addition to the inhibitors described above, it has been also reported that HIV-1 impairs T-cell activation and immunological synapse formation between infected lymphocytes and antigen-presenting cells (APCs) by Nef (negative factor) mediation [34]–[36]. One may reason that inhibitors of T-cell activation in general, such as that mediated by Nef and gp41 TMD in particular, may also inhibit the successful replication of the virions, since the magnitude of viral replication in CD4+ cells is directly linked to their activation state [37]. However, despite the fact that infection is much more efficient in activated T-cells, replication of HIV-1 and simian immunodeficiency virus (SIV) *in-vivo* occurs in T-cells that display a low activation profile as well [38]–[41]. Since we used a high concentration of gp41 TMD peptide (5–50µg/ml), we believe that the effect *in vivo* might be a modulation of the T-cell activity rather than total inhibition.

It has been reported that lymphocyte activation, through TCR ligation or other stimuli, frequently leads to homeostatic programmed apoptosis [42], [43]. Therefore, it is not unlikely that the gp41 ENV expression *in vivo* may lead to apoptosis due to TM-TM interactions of the gp41 and the TCR complex that modulate the appropriate signaling. The rapid death of the infected cells will most certainly limit the production of the virus. For that reason, in order to ensure its spread, the virus must establish a balance between the apoptosis-prone activation state and the replication-unfavorable environment of resting cells.

Although it is still unclear how these target cell effects are related to the replication efficiency of the virus, various viral proteins are known to be involved in these phenomena. For example, Nef and Tat (transcriptional transactivator) are known to modulate T-cell activity, which is likely to facilitate viral replication [35], [44]–[46]. Therefore, the fact that gp41 TMD inhibits or modulates T-cell activity does not necessarily imply that viral replication is decreased. The presence of gp41 on the cell membrane prior to and during budding may interfere with the ability of infected T cells to proliferate, thus allowing the virus to harness most cellular resources for its own needs. Hence, we suggest here that HIV-1 can utilize gp41 TM inhibitory mechanism to facilitate viral replication and to enable an efficient budding of the virions.

As we reported earlier, HIV-1 uses the FP in the N terminus of gp41 for anchoring to the target cell and to inhibit T cell activation [21], [23]. We show here that gp41 TMD interacts with CD3 and TCRα. Therefore, it is likely that gp41 TMD is also located within the TCR-FP region during the membrane fusion phase and for that reason may have a role in an efficient fusion process and as an alternative mechanism to ensure that the T cell will not be activated during this central step of infection. However, further experiments are required to better understand the mechanisms by which the virus modulates signaling within the infected cell. Note that an interesting theoretical model published recently try to explain TCR modulation by the appearance of two electro-positively charged residues within virus fusion peptides causing electrostatic disturbance within the complex [47].

It has been argued recently that HIV-1 and microvsicles from T cells share a common glycome, which may indicate a common origin [48]. Our *in silico* results pinpoints a high similarity between the gp41 TM region and the TCRα TMD region (CP domain). In addition, the similarity between gp41 TMD and TCRα CP was found to be of far greater significance than that between other virsues' TMD and TCRα CP. Therefore, the question regarding the origin of this sequence is still unclear.

In summary, the present study demonstrates a new weapon that HIV-1 uses to penetrate into the host cell and modulates its immune response. This immunosuppressive activity of gp41 TMD might be exploited in the future for the design of new therapies for autoimmune disease.

## Materials and Methods

### Ethics statement

All animal experiments were conducted at the Weizmann Institute of Science and approved by the Weizmann Institutional Animal Care and Use Committee (IACUC) according to the Israel law and the National Research Council guide (Guide for the Care and Use of Laboratory Animals 2010).

### Materials

Rink amide MBHA resin and 9-fluorenylmethoxycarbonyl (Fmoc) amino acids were purchased from Calibochem-Novabiochem AG (Switzerland). Other reagents used for peptide synthesis include N,N-diisopropylethylamine (DIEA, Aldrich), dimethylformamide, dicheloromethane, and piperidine (Biolab, IL). Egg phosphatidylcholine (PC) was purchased from Lipid Products (South Nutfield, UK). 4-chloro-7-nitrobenz-2-oxa-1,3-diazole fluoride (NBD-F) and rhodamine-*N*-hydroxysuccinimide (Rho-N) were purchased from Molecular Probes (Junction City, OR, USA). The myelin oligodendrocyte glycoprotein (MOG) p35–55 antigen used for the specific activation of the T-cell line was synthesized using the Fmoc technique with an automatic multiple peptide synthesizer (AMS 422, ABIMED, Langenfeld, Germany). The Hamster anti-mouse anti-CD3 antibody was collected by trichloroacetic acid (TCA) precipitation from 2C11 hybridoma supernatant.

### Peptide synthesis and fluorescent labeling

Peptides were synthesized using the Fmoc solid phase method on Rink amide resin (0.68 meq/gm), as previously described [49]. The synthetic peptides were purified (greater than 98% homogeneity) by reverse phase high performance liquid chromatography (RP-HPLC) on a C4 column using a linear gradient of 30–70% acetonitrile in 0.1% trifluoroacetic acid (TFA) for 40 minutes. The peptides were subjected to amino acid and mass spectrometry analysis to confirm their composition. To avoid aggregation of the peptides prior to their use in the cell culture assays, the stock solutions of the concentrated peptides were maintained in dimethyl sulfoxide (DMSO). The final concentration of DMSO in each experiment was lower than 0.25% vol/vol and had no effect on the system under investigation. For NBD-F fluorescent labeling, resin-bound peptides were treated with NBD-F (2-fold excess) dissolved in dimethyl formamide (DMF), leading to the formation of resin-bound N-terminal NBD peptides [50]. After 1 h, the resins were washed thoroughly with DMF and then with methylene chloride, dried under nitrogen flow, and then cleaved for 3 h with TFA 95%, H_2_O 2.5%, and triethylsilane 2.5%. For Rho-N fluorescent labeling, the Fmoc protecting group was removed from the N-terminus of the resin-bound peptides by incubation with piperidine for 12 min, whereas all the other reactive amine groups of the attached peptides were kept protected. The resin-bound peptides were washed twice with DMF, and then treated with rhodamine-*N*-hydroxysuccinimide (2-fold excess), in anhydrous DMF containing 2% DIEA, leading to the formation of a resin-bound *N*-rhodamine peptide. After 24 h, the resin was washed thoroughly with DMF and then with methylene chloride, dried under nitrogen flow, and then cleaved for 3 h with TFA 95%, H_2_O 2.5%, and triethylsilane 2.5%.The labeled peptides were purified on a RP-HPLC C4 column as described above. Unless stated otherwise, stock solutions of concentrated peptides were maintained in DMSO to avoid aggregation of the peptides prior to use.

### Preparation of Large Unilamellar Vesicles (LUV)

Thin films of PC were generated after dissolving the lipids in a 2∶1 (v/v) mixture of CHCL_3_/MeOH and drying them under a stream of nitrogen gas while rotating them. The films were lyophilized overnight, sealed with argon gas to prevent oxidation of the lipids, and stored at −20°C. Before the experiments, films were suspended in the appropriate buffer and vortexed for 1.5 min. The lipid suspension underwent five cycles of freezing-thawing and extrusion through polycarbonate membranes with 1- and 0.1-µm diameter pores to create large unilamellar vesicles.

### Fluorescence Energy Transfer (FRET) measurements

The FRET experiments were performed by using NBD and Rho labeled peptides. Fluorescence spectra were obtained at room temperature, with excitation set at 467 nm (10-nm slit) and emission scan at 500–600 nm (10-nm slits). In a typical experiment, a NBD-labeled peptide was added first from a stock solution in DMSO (final concentration 0.1 µM and a maximum of 0.25% (v/v) DMSO) to a dispersion of PC LUV (100 µM) in PBS. This was followed by the addition of Rho labeled peptide in several sequential doses ranging from 0.025 µM to 0.075 µM (stock in DMSO). Fluorescence spectra were obtained before and after addition of the Rho labeled peptide. The fluorescence values were corrected by subtracting the corresponding blank (buffer with the same vesicles concentration). The statistical analysis was performed using ANOVA for the pick measurements at 535nm (n = 3, * p<0.05).

### Co-localization of peptides with TCR molecules

Resting MOG35–55 T-cells or after activation for 72 h with MOG35–55 and APC were blocked with 1% BSA at room temperature to block non-specific binding. After 30 min the cells were washed and divided into aliquots containing 100,000 cells per 100 µl, and either gp41 TMD or a control membrane-binding peptide (AMP-scr) was added (final concentration of 2µM) for 1 h at 37°c. The cell were then washed and labeled with the tested antibody for 25min at RT followed by biotin-conjugated anti-hamster IgG 25min at RT and Streptavidin PE-Cy-5 10min at RT (all from eBioscience San-Diego CA, USA). Anti-hamster IgG followed by Streptavidin-Cy-5 served as a measure of non-specific binding. AMP peptide served as low TCR affinity control. The cells were analyzed by confocal fluorescence microscopy using Lab-Tek 8 chambers cover-glass (nunc) with living cells. The labeled cell samples were observed under a fluorescence confocal microscope. PE-Cy-5 excitation was done with HeNe laser 633nm (emission data was collected with filter BA660IF, 660nm long pass). NBD excitation was done with Ar laser 488nm (emission data was collected from 505–525nm). In order to quantify the co-localization percentage we utilized the “Simple PCI” software. The co-localization percentage was calculated as described below:
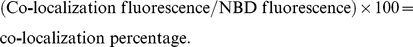



### T-cell activation and proliferation

T-cells were plated onto round 96-well plates in medium containing RPMI-1640 supplemented with 2.5% fetal calf serum (FCS), 100 U/ml penicillin, 100 µg/ml streptomycin, 50µM 2β-mercaptoethanol, and 2mM L-glutamine. We used 12×10^4^ cells of the T-cell line specific to MOG p35–55, 5×10^5^ irradiated (3000 rad) spleen cells (APC), and 10 µg/ml of MOG p35–55 were added to each well. In addition, peptide corresponding to the gp41 TM region was added. Each determination was made at least in triplicate.

In order to exclude interaction between the examined peptides and the MOG p35–55 antigen, we initially added the MOG p35–55 antigen to the APCs in a test tube, and in a second test tube we added the examined peptides to the T-cells. After 1 hour, we mixed the APCs with the T-cells and incubated them for 72h in a 96 well round bottom plate.

For some experiments, T cells were activated with immobilized anti-CD3 antibodies [51] or PMA/ionomycin as described [3]. After 72 hours, at 37°C in a 7.5% CO_2_ humidified atmosphere, the T-cells were pulsed with 1µCi (H^3^) thymidine, with a specific activity of 5.0 Ci/mmol, for 7 hours, and (H^3^) thymidine incorporation was measured using a 96-well plate beta-counter. The mean cpm ± Standard Deviation (SD) was calculated for each quadruplicate or more. The results of T-cell proliferation experiments are shown as the percentage of T-cell proliferation inhibition triggered by the antigen in the absence of gp41 TM peptide. The statistical analyses were performed using ANOVA.

### Mice

C57BL/6J mice were purchased from Harlan Olac (Bicester UK). The mice were maintained in a specific pathogen-free facility.

### Bioinformatics database analysis

To evaluate the occurrence of TCRα CP-like TMDs in viruses, a dataset of putative viral TMDs was constructed based on the viral sub-division within the Uniport Knowledge Base, consisting on the intensively annotated Swiss-Prot database (version 57.10, total of 29,252 entries) [52]. All sequences containing at least one TM annotation in the FT field were extracted from the dataset to create a library of TM viral domains, where every entry is composed from a distinct putative TMD within a protein. Overall, the library contained 6175 entries distinct at the sub-species/variants level. Entries belonging to the same taxonomic species were grouped into clusters which contained multiple sequences derived from several sub-species or variants. For our statistical analysis (Wilcoxon Rank Test) only clusters in which n>3 were used (overall 2874 entries were grouped into 265 clusters). Taxonomic clustering of results was conducted according to the tax ID lineage of each distinct entry. In the next step, a pairwise alignment of the TCRα CP was performed against each of the dataset sequences utilizing the EMBOSS package Needle pairwise global alignment [25] at the (http://www.ebi.ac.uk/Tools/emboss/align/index.html) server. Alignments parameters were set using the Blosum40 matrix with gap opening/cost of 10/10 respectively. Results were ranked according to the clusters' Z-score (normalized by the mean and standard deviation of the 265 clusters alignment scores) [53] and were analyzed using Matlab software (MathWorks, Natick,Mass). Statistical significance was determined according to the Benjamini-Hochberg method (E(FDR)<0.05) [54].
